# Assessment of psychological distress in patients with cervical dysplasia according to age, education, information acquisition and information level

**DOI:** 10.1007/s00404-024-07660-6

**Published:** 2024-08-01

**Authors:** Anne Cathrine Scherer-Quenzer, Saskia-Laureen Herbert, Tanja Schlaiss, Achim Wöckel, Joachim Diessner, Jan-Peter Grunz, Jelena Findeis, Matthias Kiesel

**Affiliations:** 1 Department of Obstetrics and Gynecology, University Hospital of Wuerzburg, Josef-Schneider-Strasse 4, 97080 Würzburg, Germany; 2 Department of Diagnostic and Interventional Radiology, University Hospital Wuerzburg, Oberduerrbacher Str. 6, 97080 Würzburg, Germany

**Keywords:** Psychological burden, Abnormal Pap smear, Risk factors

## Abstract

**Purpose:**

This study aims to investigate the psychological distress experienced by patients with an initial diagnosis of abnormal Pap smears or dysplastic changes of the cervix uteri. It investigated whether patients’ age, education, information level and approach to information acquisition have an impact on their psychological distress.

**Methods:**

A total of 364 female patients, aged 20–80 years, referred to the special dysplasia consultation hour at the Department of Obstetrics and Gynecology, Wuerzburg, completed a questionnaire containing validated items to assess information level, information acquisition, information needs and psychological distress, including a distress thermometer. Data from questionnaires and medical reports were used for analysis.

**Results:**

The study found that 56.9% of patients experienced psychological distress before their first visit. Patients under 44 years of age, especially those with concerns about fertility and sexuality, and those with lower levels of education showed higher levels of distress (*p*-value = 0.018 and *p*-value = 0.037). 40.9% of patients felt poorly informed and 53.7% of patients wanted more information before their visit. Correlational analysis showed that the method of obtaining information correlated with the desire for more information (*p*-value < 0.001). Those who received information via the Internet felt less informed, wanted more information and experienced more anxiety.

**Conclusion:**

These findings highlight the need for improved patient education strategies and effective doctor-patient communication to address the knowledge gap and reduce patient distress. In addition, healthcare providers should ensure that patients have access to reliable online resources for accurate information.

## What does this study add to the clinical work

**Table:** 

This study highlights the prevalence of psychological distress among patients with cervical dysplasia and investigates the extent to which factors such as age, information acquisition, level of information, desire for more information and educational level have an influence on patients’ psychological distress. These findings can help tailor patient education strategies and improve doctor-patient communication and overall quality of care for patients undergoing cervical screening and diagnosis.	

## Introduction

In 2022 cancer was diagnosed in 19.98 million people worldwide [1]. It is well known that cancer diagnosis is associated with psychological distress [2]. The extent of psychological comorbidity in cancer patients was first scientifically investigated in the 1970s and 1980s. Since then, the scientific focus on this topic has increased [3]. A comparative study of oncology patients, chronically ill patients and healthy people showed that 20% of oncology patients described themselves as psychologically distressed, compared with 10.6% of healthy people and 14.8% of chronically ill people. The risk of subsequent mental disorders is particularly high in young long-term survivors [4].

One type of cancer that particularly affects young women is cervical cancer. According to recent data, it is the 14th most common cancer and the 4th most common cancer in women worldwide [5]. In a study conducted in the USA in 2005, patients with an average age of 45 years who were being followed for 5–10 years after treatment for cervical cancer were asked about their diagnosis. The patients were healthy at the time of the survey and reported a good quality of life. However, 69% said they would have attended a support group if it had been offered at the time of diagnosis [6]. A study conducted in Germany in 2015 with 595 patients found that after being informed of an abnormal Pap smear, 68.8% of women reacted with anxiety and 26.3% reacted with panic. For 63.9% of patients, talking to their doctor was the most important source of information. But even after the discussion, 51.4% were still worried and only 24.4% were reassured [7].

The aim and interest of this study is, firstly, to investigate the psychological distress experienced by patients with abnormal Pap smears or dysplastic changes of the cervix. The second aim is to determine whether age, education and the way in which information is obtained have an influence on the psychological distress of patients, to optimise and support patient care, patient education and patient information in the future.

## Methods

### Human patient data

This retrospective study was registered with the Institutional Review Board (Ref. No. 20210628 01).

The female patients were referred to the special dysplasia consultation hour at the Department of Obstetrics and Gynecology (University of Wuerzburg) by their respective gynecologists in case of abnormal Pap smears or HPV positivity for further diagnostics and treatment. During their initial visit, the patients were given the questionnaire found in the Appendix. In addition, information was extracted from each patient’s medical report.

The questions are based on a validated questionnaire for assessing psychological distress in patients with cervical dysplasia (Cervical Dysplasia Distress Questionnaire = CDDQ), which was validated by Shinn et al. in a study involving 661 participants [8]. The CDDQ was supplemented with four questions addressing background information of the affected individual and the Distress Thermometer. The Distress Thermometer is a developed screening instrument from the National Comprehensive Cancer Network to measure psychosocial distress in oncologically ill patients. The thermometer allows patients to indicate their psychological burden on a numerical scale from 0 to 10.” 0 “represents” “no burden” and “10” represents “very burdened.” Over the years, the thermometer has been validated through numerous studies [9].

The four additional questions related to the level of education, the subjective level of information, the desire for further information, the method of obtaining information about dysplasia, and the adequacy of prior information provided by the personal gynecologist. These four questions were chosen to specifically assess the patients’ level of knowledge and then to discuss possible ways of optimising education and information in the future. Each patient could choose up to five answers.

Pap smears were interpreted by two experienced gynecological cytologists with more than 20 years of experience at the Department of Obstetrics and Gynecology (University of Wuerzburg). HPV testing was evaluated by polymerase chain reaction (PCR) amplification followed by genotyping at the Institute of Virology and Immunology (University of Wuerzburg). The genotypes were categorised as 'high risk' and 'low risk'.

### Statistical analysis

All variables were documented in an Excel file (Microsoft Office Excel 2013, Washington, USA). The data was analyzed using the statistical software SPSS (Version 24) from IBM (SPSS Inc., Chicago, IL. USA) and the following statistical test procedures:

-Descriptive data analysis.
-Correlation analysis using Spearman's rank correlation coefficient (rho).
-Mann–Whitney *U* test.
-Chi-squared test.
-Kruskal-Walis test

*p*-values of < 0.05 were considered statistically significant.

### Use of large language models

ChatGPT Version 4.0 (OpenAI Inc., San Francisco) was used for language quality check.

## Results

### Patient cohort

The patient cohort consisted of a total of 364 female patients aged between 20 and 80 years, with a mean age of 43.3 years (SD = 11.3). Of the Pap tests performed, Pap IVa-p was the most common (25.5%), followed by Pap IIID2 (21.4%) and IIp (21.2%). In addition, 70.3% of patients had a high-risk HPV subtype. Patients’ overall psychological distress was assessed using the Distress Thermometer [9]. For analysis, responses from 1 to 5 were grouped as “not distressed” and responses from 6 to 10 were grouped as “distressed”. 56.9% of patients reported psychological distress (Table 1).

**Table 1:** Patient characteristics

	Mean (SD) or number (percentage)
Age in years	43.3 (11.3)
Pap IVa-p	93 (25.5)
Pap IIID2	78 (21.4)
Pap IIp	77 (21.2)
Others	116 (31.9)
HPV high risk	256 (70.3)
HPV low risk	13 (3.6)
HPV negativ	32 (8.8)
No data	63 (17.3)
Low distress level	157 (43.1)
High distress level	207 (56.9)

SD = standard deviation. HPV high risk = 16, 18, 31, 33, 35, 39, 45, 51, 52, 56, 58, 59, HPV low risk = 40, 42, 43, 44, 54, 61, 72, 81, low distress level measured from 1 to 5 (according to Distress Thermometer), high distress level measured from 6 to 10 (according to Distress Thermometer)

### Psychological burden and information acquisition based on patients’ age

The following results aim to illustrate how the patients’ age influences their psychological burden and concerns. The cohort was divided into a group of younger and older women according to the mean age of the group of 43.3 years, which was used as a threshold between “young” and “old”. While 57.3% of the younger women reported psychological distress, only 42.8% of the older patients reported the same (*p*-value = 0.018). There was also a statistically significant difference in information seeking between younger and older patients (*p*-value = 0.028). Among younger patients, 62.1% sought information from their gynecologist, compared to 74.8% of older patients. 27.2% of younger patients obtained information only from the Internet, compared to 14.8% of older patients. Although most patients in both groups were generally informed by the attending gynecologist (67.6%), statistical analysis shows that younger women were significantly more likely than older women to obtain their information only from the internet, and older women were significantly more likely than younger women to obtain their information only from their gynecologist (Table 2).

**Table 2:** Information acquisition based on patients’ age

Age		Young (< 43.3 yr)	Old (≥ 43.3 yr)	Sum
No information acquisition	Observed count	19	12	31
	Expected count	17.7	13.3	31
	Proportion within the group (in%)	9.2	7.7	8.6
	R	0.3	−0.4	
Information through gynecologist	Observed count	128	116	244
	Expected count	139.2	104.8	244.0
	Proportion within the group (in%)	62.1%	74.8%	67.6%
	R	−1	1.1	
Information through internet (solely)	Observed count	56	23	79
	Expected count	45.1	33.9	79.0
	Proportion within the group (in%)	27.2%	14.8%	21.9%
	R	1.6	−1.9	
Information through family/friends	Observed count	3	4	7
	Expected count	4.0	3.0	7.0
	Proportion within the group (in%)	1.5%	2.6%	1.9%
	R	−0.5	0.6	

Cross tabulation and Chi-square test. *R* = Standardized residual

### Psychological burden based on information level

Question 1 assessed the patients’ level of information during their initial visit. Patients could select their answers on a numerical scale from 1 to 10. For the analysis of Question 1 responses from 1 to 5 were grouped as “poorly informed”, and responses from 6 to 10 were grouped as “well informed”. The distribution of Question 1 revealed that 40.9% of the patients felt poorly informed. These patients also reported a higher psychological burden (based on the Distress Thermometer) compared to others (*p*-value = 0.048).

### Psychological burden based on information acquisition

Question 2 examined where patients had obtained information prior to their first visit. The response options for question 2 were ‘not at all’, ‘from the gynecologist’, ‘from the internet’ or ‘from friends/acquaintances’. Multiple answers were possible for this question. Overall, 67.6% of patients obtained information from their gynecologist. The second most common source was the internet with 21.9%. Only 8.6% of patients had received no information before their first visit (Table 2). While 62.3% of the women who had obtained information from the internet were psychologically distressed, 57.8% of those who had been informed by their gynecologist reported the same (Table 3). Correlation analysis shows that the level of psychological distress does not depend on the way in which the information was obtained (*p*-value = 0.971).

**Table 3:** Cross table concerning information acquisition and measured distress level

		Distress-level	1	2	3	4	5	6	7	8	9	10
No information acquisition	Observed count		1	4	4	4	3	0	3	3	1	2
	Percentage		4	16	16	16	12	0	12	12	4	8
Information through gynecologist	Observed count		14	16	15	11	19	8	28	24	25	18
	Percentage		7.9	9.0	8.4	6.2	10.7	4.5	15.7	13.5	14	10.1
Information through internet (solely)	Observed count		8	10	5	9	11	8	13	17	19	14
	Percentage		7.0	8.8	4.4	7.9	9.6	7.0	11.4	14.9	16.7	12.3
Information through family/friends	Observed count		1	1	0	0	3	2	2	5	3	1
	Percentage		5.6	5.6	0	0	16.7	11.1	11.1	27.8	16.7	5.6
Sum			20	26	22	20	28	14	40	36	33	27

Distress level measured from 1 to 10. Low distress level = 1–5. High distress level = 6–10

Correlation between information acquisition, wish for information and information level.

Question 3, which inquired about the desire for information before the visit, also had ten response options. For the analysis, responses from 1 to 5 were considered “no further information desired”, and responses from 6 to 10 were considered “desire for more information”. 53.7% of the patients wished for more information before their visit. Further analysis indicated that the desire for more information correlates with the form of information acquisition (*p*-value < 0.001). While 63.6% of the women who had obtained information from the internet wished for more information, only 47.0% of those informed by their gynecologist reported the same (Table 4). This is underscored by the fact that only 35.3% of the patients informed by their gynecologist felt poorly informed while 45.8% of those obtaining information by the internet reported the same (Table 5).

**Table 4:** Cross table concerning information acquisition and wish for more information

		Wish for information	1	2	3	4	5	6	7	8	9	10
No information acquisition	Observed count		7	1	4	2	3	0	3	4	2	4
	Percentage		23.3	3.3	13.3	6.7	10	0	10	13.3	6.7	13.3
Information through gynecologist	Observed count		45	21	21	7	34	18	25	30	8	33
	Percentage		18.6	8.7	8.7	2.9	14	7.4	10.3	12.4	3.3	13.6
Information through internet (solely)	Observed count		12	6	8	5	25	10	17	26	13	32
	Percentage		7.8	3.9	5.2	3.2	16.2	6.5	11	16.9	8.4	20.8
Information through family/friends	Observed count		2	0	0	0	6	3	4	2	3	5
	Percentage		8	0	0	0	24	12	16	8	12	20
Sum			57	23	28	12	45	24	35	48	18	65

With for more information measured from 1 to 10. No further information desired = 1–5. Desire for more information = 6–10

**Table 5:** Cross table concerning information acquisition and information level

		Information Level	1	2	3	4	5	6	7	8	9	10
No information acquisition	Observed count		4	1	4	2	5	2	4	4	2	2
	Percentage		13.3	3.3	13.3	6.7	16.7	6.7	13.3	13.3	6.7	6.7
Information through gynecologist	Observed count		7	10	9	12	48	15	31	47	25	40
	Percentage		2.9	4.1	3.7	4.9	19.7	6.1	12.7	19.3	10.2	16.4
Information through internet (solely)	Observed count		4	11	14	12	29	15	19	24	14	11
	Percentage		2.6	7.2	9.2	7.8	19	9.8	12.4	15.7	9.2	7.2
Information through family/friends	Observed count		0	1	1	3	4	3	3	5	2	3
	Percentage		0	4	4	12	16	12	2	20	8	12
Sum			15	21	25	21	63	27	40	61	36	48

Information level measured from 1 to 10. Poorly informed = 1–5, Well informed = 6–10

### Psychological burden based on patients’ education level

Question 4 of the questionnaire aimed to assess the education level of the patients. The response options were “no school degree,” “vocational training” or “general matriculation standard”. The analysis of question 4 showed that 5.1% of the patients had no school degree, 61.1% had vocational training, and 33.5% possessed a general matriculation standard. To investigate whether the type of information acquisition depended on the education level, the distribution of information acquisition was calculated separately for different education categories. Overall, patients with vocational training and a general matriculation standard tended to obtain information from more than one source. There was a negative significant correlation between patient’s overall insecurity and their education level (*p*-value = 0.038), indicating that patients with lower education levels were more insecure compared to well-educated patients.

### Possible reasons for increased psychological distress and the effects of increased psychological distress

Younger women have a greater concern compared to older women about not being able to have children anymore (*p*-value < 0.001). Regardless of age, women who wished for more information beforehand are significantly more concerned about developing cancer (*p*-value < 0.001). Women exhibiting increased psychological distress experienced the examination as significantly more unpleasant than patients who were well-informed (*p*-value < 0.001).

## Discussion

This study aimed to investigate the extent of psychological distress in patients with abnormal Pap smears and the factors that influence this distress.

We were able to demonstrate that nearly 60.0% of the patients already experienced psychological distress during their initial visit to our special dysplasia consultation hour at the Department of Obstetrics and Gynecology (University of Wuerzburg). In 2020, Jentschke et al. conducted a survey concerning the mental state of over 2000 women, who had to attend follow-up appointments over an extended period due to abnormal Pap smears. For many women, the uncertain situation with prolonged waiting times between appointments caused significant distress. The proportion of psychologically distressed individuals was 70.0%, which aligns with the findings of our study [10]. This is a considerable percentage, taking into consideration that an abnormal Pap smear affects several million women annually in Germany. It underscores the importance of comprehensive education and providing informational materials.

When considering the age of the affected women it is noteworthy that many women experience abnormal findings at a young age. In 2020, one-third of new cases of cervical carcinoma occurred in women under 44 years of age [11]. In the analysis of the collected questionnaire data, half of the patients with abnormal Pap smears were younger than 44 years old. As expected, the younger group of patients, especially those with concerns about fertility and sexuality, experiences greater distress. These results are consistent with the findings of Jentschke et al. in 2020, which indicated that abnormal Pap smears particularly lead to high distress and affect family planning for women with existing fertility desires.

Patients’ physical and emotional burden is attributed not only to the threat of cancer development but also to the invasive nature of treatment procedures [12, 14]. For patients with histologically confirmed CIN II/III excisional treatments such as loop electrosurgical excision procedure (LEEP) and destructive treatments like ablation are common. Treatment for CIN I/II lesions is recommended after 24 months of persistence or in specific cases such as psychological stress or high-risk conditions as for instance Human immunodeficiency virus (HIV) [13]. Despite its high success rates, LEEP, requiring local or general anesthesia, is associated with invasiveness, an increased risk of major bleeding, reduced fertility and pregnancy complications such as premature delivery and increased cesarean section rates [14, 15]. To address these concerns, noninvasive options like argon plasma coagulation (APC) and Non-Invasive Physical Plasma (NIPP) offer a tissue-preserving approach with encouraging results in treating precancerous lesions [14–16]. Current studies show promising outcomes, including improved remission rates and reduced high-risk HPV status post-NIPP treatment, underscoring its potential as a safer alternative, especially for young women desiring fertility preservation [14]. Klügel et al. investigated the correlation between treatment type and psychosocial well-being post-treatment. In terms of physical Health-Related Quality of Life (HRQoL) and coping strategies, conservative therapy demonstrated higher scores and better outcomes. Thus, it must be considered, that the treatment approach can influence the psychosocial well-being of CIN II patients [17]. It might be inferred from this that less invasive methods, in comparison to LEEP, might also lead to an enhanced psychological condition. Yet, further research is needed. Moreover, non-invasive methods, which can be perceived as less uncomfortable and have fewer side effects [18] may lead to improved patient compliance with follow-up screenings and treatments, ultimately enhancing long-term outcomes. While non-invasive procedures offer advantages in terms of reduced invasiveness, preservation of fertility, and decreased risk of complications, one limitation is that some procedures do not provide histologic tissue. This can have implications for diagnostic accuracy, treatment monitoring, and follow-up care. Consequently, patient preferences, individual risk factors, and the expertise of healthcare providers should also be taken into account when determining the most suitable treatment approach for each patient.

Regarding information acquisition according to age, both groups primarily obtained information from their gynecologist. However, the internet plays a more important role as an information source for the younger group, which may lead to a higher risk of misinformation [19].

Based on the education distribution, it can be observed that patients with lower education levels generally experience higher distress. However, as seen above, the younger, better-educated group of women exhibited higher average psychological distress than the older patients.

Overall, over 40% of patients reported feeling poorly informed. This is a substantial portion, considering that patients had already been informed about the abnormal findings by their gynecologist. Patients are further referred to the clinic and have the opportunity to gather additional information before their visit to our consultation hour. As mentioned in the introduction, despite the national HPV vaccination campaign and increased availability of information and education, many patients still find the provided information inadequate [19].

It should be acknowledged that variations in patient education by their respective physician can affect patient knowledge. When examining the information available on the internet, it becomes clear that most health insurance companies highlight key points about screening on their websites and brochures from the Joint Federal Committee (Gemeinsamer Bundesausschuss) are also available. The brochure from the Joint Federal Committee contains all the necessary information regarding early detection, basic information on cervical carcinoma, the procedure of examinations, and the significance of examination results. Additionally, it explains the pros and cons of early detection. These brochures are available for free download. A study conducted in the USA in 1993 demonstrated the effect of targeted information and education regarding abnormal Pap smears before a clinic visit. Patients in this study were given the choice of receiving informational materials before their appointment. Those who received the materials reported significantly less concern about the abnormal Pap smear and the risk of cancer in subsequent questionnaires compared to the cohort that did not receive the materials [20]. Another study conducted in 1994 provided evidence that patients who received informational brochures were significantly more likely to attend follow-up appointments after abnormal Pap smears compared to those who did not receive informational materials [21]. The desire for more information is evident, with over 50% of patients wishing for more information before their visit. Furthermore, since over 68% of patients primarily obtain information from their gynecologist, with the internet being the second most common source, it underscores the importance of the doctor-patient relationship.

Taken together, we can summarize that patients who obtained information from the internet reported feeling less informed, had a greater desire for information before their visit, and experienced greater distress. These results, coupled with the fact that the gynecologist remains the primary source of information for patients, emphasizes how crucial adequate education and the provision of informational materials from treating doctors are.

## Conclusion

This study shows the significant psychological distress faced by patients with abnormal Pap smears or cervical dysplasia. Factors that influence psychological stress include age, education and information acquisition. Age and education level emerged as defining factors in distress levels. Younger patients, especially those under 44 years, experience heightened distress due to concerns about fertility and future health. Patients with lower education levels tend to experience higher distress. The age and education-related variations in information sources and preferences underline the importance of tailoring educational resources to different age groups. Furthermore, it is important to ensure the accuracy of online health information as it seems that numerous patients are not fully utilizing reliable informational resources. Thus, this study underlines the need for improved patient education and the dissemination of accurate information by healthcare providers to alleviate the psychological distress experienced by patients with abnormal Pap smears.

## Data Availability

The datasets generated during and/or analysed during the current study are available from the corresponding author upon reasonable request.

## Appendix

### Questionnaire 1

Dear patient,

Please fill out the following questions before the examination:

Question 1 and 3 can be answered by selecting a number on a scale from 1 to 10. The other questions should be answered by selecting the statement that applies. The data will be anonymized.

1. To what extent do you feel informed about your visit today. 1 corresponds to “very poor”, 10 corresponds to “very well” 1 2 3 4 5 6 7 8 9 10.
2. How did you inform yourself for today’s visit?
  - Not at all.
  - From the gynaecologist.
  - From the internet.
  - From friends/acquaintances.
3. Would you have liked more information in advance? 1 corresponds to “no further information”, 10 corresponds to “more information” 1 2 3 4 5 6 7 8 9 10.
4. Please select the level of education that applies to you.
  - No school degree.
  - Vocational training.
  - General matriculation standard.

### Cervical dysplasia distress questionnaire

#### Tension and discomfort

Did you find the exams uncomfortable?

Did you find the exams emotionally upsetting?

Did the exams make you nervous?

Did the exam hurt?

Did you feel tense?

Were you nervous?

#### Embarassement

Were you uncomfortable being partly undressed?

Were you embarrassed having your private parts touched by the doctors or the nurse?

#### Sexual and reproductive consequences

How worried are you that you would lose your chance to have a baby?

Have you been worried that you could give the problem to a sexual partner.

Have you been worried whether a sexual partner will think they can catch the problems from you.

Have you been worried whether you should continue having sex?

Have you been worried that this problem might affect how attractive you are to your sexual partner?

Have you been worried whether having sex will make the problem worse?

Have you been worried whether others think you have had more sexual partners than you should?

Have you been worried about sex being more painful now?

Have you been worried that this problem might affect how much you enjoy sex?

#### Health consequences

How worried are you that cancer will appear in your body?

Have you worried about the test results?

Have you worried that you may have cancer?

How worried are you that you might die?

Have you been worried that your problem may turn into cancer?

How worried are you that you might die from cervical cancer?

#### Distress Thermometer

Please circle on the thermometer the number that describes best how stressed you have felt in the past week, including today?

1 2 3 4 5 6 7 8 9 10
