# Correction: Assessment of psychological distress in patients with cervical dysplasia according to age, education, information acquisition and information level

**DOI:** 10.1007/s00404-025-08011-9

**Published:** 2025-04-15

**Authors:** Anne Cathrine Scherer-Quenzer, Saskia-Laureen Herbert, Tanja Schlaiss, Achim Wöckel, Joachim Diessner, Jan-Peter Grunz, Jelena Findeis, Matthias Kiesel

**Affiliations:** 1 Department of Obstetrics and Gynecology, University Hospital of Wuerzburg, Josef-Schneider-Strasse 4, 97080 Würzburg, Germany; 2 Department of Diagnostic and Interventional Radiology, University Hospital Wuerzburg, Oberduerrbacher Str. 6, 97080 Würzburg, Germany

**Correction: Archives of Gynecology and Obstetrics (2024) 310:2173–2181** 10.1007/s00404-024-07660-6

The article “Assessment of psychological distress in patients with cervical dysplasia according to age, education, information acquisition and information level”, written by Anne Cathrine Scherer‑Quenzer · Saskia‑Laureen Herbert · Tanja Schlaiss · Achim Wöckel · Joachim Diessner · Jan‑Peter Grunz · Jelena Findeis and Matthias Kiesel was originally published under exclusive license to The Author(s), under exclusive licence to Springer-Verlag GmbH Germany, part of Springer Nature 2024. As a result of the subsequent decision to publish the article under the open access model, the article’s copyright notice was changed on 1 March 2025 to © The Author(s) 2025 and the article is now distributed under a Creative Commons Attribution 4.0 International License, which permits use, sharing, adaptation, distribution and reproduction in any medium or format, as long as you give appropriate credit to the original author(s) and the source, provide a link to the Creative Commons licence, and indicate if changes were made. The images or other third party material in this article are included in the article’s Creative Commons licence, unless indicated otherwise in a credit line to the material. If material is not included in the article’s Creative Commons licence and your intended use is not permitted by statutory regulation or exceeds the permitted use, you will need to obtain permission directly from the copyright holder. To view a copy of this licence, visit http://creativecommons.org/licenses/by/4.0.

